# Regulation of human salt-sensitivite hypertension by myeloid cell renin-angiotensin-aldosterone system

**DOI:** 10.3389/fphys.2023.1208270

**Published:** 2023-07-18

**Authors:** Lale A. Ertuglu, Ashley Pitzer Mutchler, Fernando Elijovich, Cheryl L. Laffer, Quanhu Sheng, Celestine N. Wanjalla, Annet Kirabo

**Affiliations:** ^1^ Department of Medicine, Division of Nephrology, Vanderbilt University Medical Center, Nashville, TN, United States; ^2^ Department of Medicine, Division of Clinical Pharmacology, Vanderbilt University Medical Center Nashville, Nashville, TN, United States; ^3^ Department of Biostatistics, Vanderbilt University Medical Center, Nashville, TN, United States; ^4^ Department of Internal Medicine, Division of Infectious Diseases, Vanderbilt University Medical Center Nashville, Nashville, TN, United States

**Keywords:** salt sensitivity, renin-angiotensin-aldosterone system, myeloid immune cells, hypertension, immunity

## Abstract

**Introduction:** Salt sensitivity of blood pressure is a phenomenon in which blood pressure changes according to dietary sodium intake. Our previous studies found that high salt activates antigen presenting cells, resulting in the development of hypertension. The mechanisms by which salt-induced immune cell activation is regulated in salt sensitivity of blood pressure are unknown. In the current study, we investigated dietary salt-induced effects on the renin-angiotensin-aldosterone system (RAAS) gene expression in myeloid immune cells and their impact on salt sensitive hypertension in humans.

**Methods:** We performed both bulk and single-cell sequencing analysis on immune cells with *in vitro* and *in vivo* high dietary salt treatment in humans using a rigorous salt-loading/depletion protocol to phenotype salt-sensitivity of blood pressure. We also measured plasma renin and aldosterone using radioimmunoassay.

**Results:** We found that while *in vitro* high sodium exposure downregulated the expression of renin, renin binding protein and renin receptor, there were no significant changes in the genes of the renin-angiotensin system in response to dietary salt loading and depletion *in vivo*. Plasma renin in salt sensitive individuals tended to be lower with a blunted response to the salt loading/depletion challenge as previously reported.

**Discussion:** These findings suggest that unlike systemic RAAS, acute changes in dietary salt intake do not regulate RAAS expression in myeloid immune cells.

## Introduction

Salt-sensitivity of blood pressure, a phenotype in which high dietary salt intake leads to increase in blood pressure, affects approximately half of people with hypertension (Weinberger et al., 1986) and is associated with increased risk of cardiovascular disease (Weinberger et al., 2001). Despite the high prevalence and prognostic implications of salt sensitivity, the underlying mechanisms remain unclear.

Recent studies have established that immune system and inflammation are important in the development of salt-sensitive hypertension and associated end-organ damage (Wenzel et al., 2011; Foss et al., 2017; Patrick et al., 2021). In our previous studies, we found that antigen-presenting cells (APCs), including dendritic cells and monocytes, are activated by extracellular high-sodium concentrations. Sodium entry into APCs via epithelial sodium channel (ENaC) leads to activation of Nicotinamide adenine dinucleotide phosphate (NADPH) oxidase and production of isolevuglandins (IsoLGs), which are highly reactive products of lipid peroxidation. Rapid adduction of IsoLGs to proteins forms neo-antigens that activate APCs, triggering the secretion of pro-inflammatory cytokines, inflammation and subsequent development of hypertension (Barbaro et al., 2017).

Renin-angiotensin-aldosterone system (RAAS) is a crucial regulator of blood pressure through its effects on renal sodium reabsorption, vascular tone and sympathetic output, and is also implicated in salt-induced blood pressure response (Shimosawa, 2013). Moreover, multiple components of the RAAS signaling pathway have been shown to directly affect immune cell activation and function (Crowley and Rudemiller, 2017; Ferreira et al., 2021). Angiotensin II directly upregulates many pro-inflammatory genes such as nuclear factor-κB, interleukin-6, monocyte chemoattractant protein-1 and induces inflammatory cell recruitment and infiltration in tissue, which drives end-organ damage in many chronic diseases including hypertension (Geisberger et al., 2015; Crowley and Rudemiller, 2017). Aldosterone has also been shown to increase the expression of adhesion molecules and pro-inflammatory mediators (Rocha et al., 2002) and trigger monocyte and macrophage infiltration into tissue (Tostes et al., 2002; Terada et al., 2012). Renin activates (pro)renin receptor expressed in human monocytes and T cells as well as vasculature that may enhance proinflammatory cytokine production (Feldt et al., 2008; Geisberger et al., 2015). Whether regulation of cellular RAAS signaling pathways play a role in myeloid immune cell activation in salt sensitive hypertension remains unknown.

In the present study, we investigated RAAS-related gene expression profiles in human monocytes collected from healthy people in response to sodium *in vitro*. We also assessed *in vivo* changes in the expression of RAAS-related genes in myeloid immune cells from people with hypertension in response to dietary salt loading and depletion based on salt sensitivity.

## Materials and methods

### Study population

Nine subjects with hypertension were recruited at the Vanderbilt University Medical Center (VUMC) from 2019 to 2021. Controls were recruited using VUMC e-mail distribution lists, Researchmatch.org, flyers in the clinics and electronic health record-based messages. The study was approved by the Institutional Review Board and all subjects signed written informed consent before their participation. Subjects between ages 18 to 65, either on antihypertensive therapy or with systolic blood pressure (SBP) > 140 mm Hg or diastolic blood pressure (DBP) > 90 mm Hg were included. Exclusion criteria included diabetes mellitus, confirmed or suspected renal, renovascular, or endocrine causes of secondary hypertension, treatment with agents known to increase BP (e.g., adrenergic agonists for attention deficit hyperactivity disorder, selective serotonin reuptake inhibitors and serotonin and norepinephrine reuptake inhibitors, chronic use of decongestants or non-steroidal anti-inflammatory drugs), treatment with agents known to modulate immune response (e.g., glucocorticoids, immunosuppressants, direct immunomodulators), current excess alcohol or illicit drug use, active or ongoing infectious or inflammatory disease (i.e., active infection or connective tissue disorder), active or ongoing cancer or a history of an acute cardiovascular event within 6 months of the study, and being pregnant. Demographic and clinical data were collected from the subjects and by chart review.

### Study protocol

During the screening visit, a physical exam was conducted, and blood pressure was measured. Prior to the study visit, all subjects stopped taking antihypertensive medications for a minimum of 2 weeks and were advised to maintain their usual diet during this time. For safety purposes, participants who stopped taking antihypertensive medications had their blood pressure measured twice a day while seated, after resting for 5 min.

The subjects were admitted to the VUMC Clinical Research Center for a period of three nights in order to determine their salt sensitivity. This was achieved through an inpatient protocol involving salt loading and depletion, which has been described in detail previously (Laffer et al., 2003). As recommended by the AHA Scientific Statement, salt loading preceeded by salt depletion, which aims to suppress renin angiotensin system uniformly in all patients, and therefore reduce baseline variability (Elijovich et al., 2016). On the night of admission, subjects were given a regular dinner and rested. The following morning, blood pressure recording was initiated using ambulatory BP monitors (Spacelabs 90,207) and baseline blood samples were obtained at 8 a.m. before any intervention. Salt loading (Day 1) was achieved by administering a diet containing 160-mEq NaCl and 70-mEq of K^+^, prepared by the University of Alabama Bionutrition Core of the Clinical Research Unit Metabolic Kitchen, along with a 2-L intravenous infusion of normal saline, given from 8 a.m. to 12 PM. Blood sampling was repeated at 8 a.m. next day (day 2) to reflect the salt-loaded state.

Salt depletion was achieved by administering three 40-mg doses of oral furosemide and a diet containing 10-mEq NaCl and 70-mEq of K^+^, on day 2. The furosemide doses were given at 8 a.m., 12 p.m., and 4 p.m. The first sample was collected from 8 a.m. to 8 p.m. to reflect the effects of furosemide, and the second was collected from 8 p.m. on day 2–8 a.m. on day 3 to reflect salt depletion. A third set of blood samples was obtained at 8 a.m. on day 3 to reflect the salt-depleted state. The subjects were discharged after blood collection on the morning of day 3.

The subjects had unlimited access to water throughout the study; however, food intake was limited to the diet provided according to the protocol. Body weights were recorded at baseline and daily at 7 a.m. Body mass index (BMI) was calculated as weight in kg divided by height in m^2^.

BPs were measured with a mercury sphygmomanometer and appropriate size cuff at the screening visit, automated monitor (Omron HEM-907XL) at home during the 2 weeks before admission for safety, and ambulatory monitor (Spacelabs 90,207) during admission. BP and pulse rate were recorded every 15 min from 6 a.m. to 10 p.m. in the daytime and every 30 min in the nighttime. The average of the BP recordings from 6 a.m. to 8 a.m. on day 1 was used as baseline BPs. The BPs recorded from 12 p.m. to 10 p.m. on days 1 and 2 were used to calculate the average BPs on salt loading and depletion, respectively.

Salt sensitivity index was defined as the mmHg change in average daytime SBP from salt loading to depletion and was assessed as a continuum. Accordingly, a bigger decrease in SBP from salt loading to depletion denoted a greater salt sensitivity index. For representation purposes, patients were divided into tertiles based on salt sensitivity index. Three subjects with the highest salt sensitivity index were represented as salt sensitive and three subjects with the lowest salt sensitivity index were represented as salt resistant.

### Laboratory analysis

Blood counts, electrolytes and creatinine, plasma renin concentration, and aldosterone were analyzed at the VUMC Pathology Laboratory. Plasma renin direct renin/renin mass and plasma aldosterone were measured using chemiluminescent radioimmunoassay. Blood was collected in EDTA tubes, centrifuged in room temperature and separated immediately. Sample collection was done after patients were in supine position for at least 15 min.

### Single cell RNA sequencing

Single-cell sequencing was performed on peripheral blood mononuclear cells (PBMCs) collected from nine subjects and batched by treatment as baseline, salt loading (Day 1) or salt depletion (Day 2). Cell hashing was used to distinguish each participant and Cellular Indexing of Transcriptomes and Epitopes by Sequencing (CITE-seq) analysis was used to profile changes in immune cells with each treatment day.

PBMCs were isolated as described by the manufacturer’s protocol (Fisher Scientific, Cat# 14-959-51D) in BD Vacutainer^®^ CPT™ Mononuclear Cell Preparation tubes. Oligonucleotides were ligated to antibodies used to stain cells by click chemistry. In cell hashing, barcodes (hashtags) specific for samples were included for antibody identification and a handle was used for polymerase chain reaction (PCR) amplification. The cell-antibody-oligonucleotide complex were pushed into a microfluidic system along with custom beads conjugated with poly dT and nucleotide sequences designed to capture the hgRNA barcodes. Oil was used to create a small vesicle of a single bead and a single cell with an antibody-oligonucleotide complex bound to its surface. The cell was lysed, and then cDNA of intracellular mRNA origin and Antibody Derived Tags (ADTs) were formed using reverse transcription of the mRNA to cDNASequencing in the Vanderbilt Technologies for Advanced Genomics (VANTAGE) facility.

A reference genome index was built using 10x Genomics Cell Ranger 6.0.2 with map reads to a reference genome (GRCh38-2020-A) and quantify genes. In-house scripts were utilized for demultiplexing sample-specific hashtags. In each hashtag of each sample, the hashtag abundance cutoff of positive cells was determined by modified R package cutoff [https://github.com/shengqh/cutoff]. Subsequently, each cell was classified as singlet with specific hashtag, doublet or negative. Then, genotype-based demultiplex result from Souporcell were integrated with this hashtag-based demultiplex result (Heaton et al., 2020). In further anlaysis, 88,387 out of 94,137 singlet cells with between 200 and 5,000 unique genes, more than 500 unique read counts, and maximum mitochondrial content of 10% were filtered in. Clustering analysis was performed via Seurat (Butler et al., 2018) with resolution 1.0. Cell type of each cluster was classified based on cell activity database (Butler et al., 2018; Franzén et al., 2019) and then manually refined based on cell type specific marker gene expression. The FindAllMarkers function of Seurat package with default Wilcoxon Rank Sum test was used to generate marker genes. edgeR (Robinson et al., 2010) was utilized to identify differential expression across conditions. The WebGestaltR (Liao et al., 2019) package was used to perform Genome Ontology and KEGG pathway over-representation analysis on differentially expressed genes. Gene set enrichment analysis was done by GSEA (Subramanian et al., 2005) package.

### Bulk RNA sequencing

Blood samples from eleven healthy subjects was used to examine changes in monocyte expression of RAAS-related genes in response to sodium *in vitro*. Subjects were 18–75 years old with no history of inflammatory disease, and the demographics of these subjects have been published elsewhere (Ruggeri Barbaro et al., 2021). Briefly, PBMCs were isolated using the Ficoll-gradient. Heparinized blood was diluted 4-fold with PBS + 2 mM EDTA buffer and layered over 15 mL of Ficoll-PaqueTM in a 50 mL conical tube. After aspirating the top plasma layer following 30 min of centrifugation at 400 g at 20°C, the layer with PBMCs was transferred to another conical tube. This tube was filled with buffer and centrifuged again at ×300 g for 10 min. To isolate monocytes, the cell pellet was resuspended. Magnetic labeling and negative selection was performed using the Miltenyi monocyte isolation kit (Miltenyi Biotec, Cat# 130-091-151) according to the manufacturer’s protocol using LS columns. Monocytes were then cultured in 12-well plates at 1 × 106/mL density in either high salt RPMI media (190 mM) or normal salt RPMI media (150 mM) for 72 h. RPMI media 1,640 (Gibco) was added with 10% FBS, 1% pen/strep, 1% HEPES, and 2-Mercaptoethanol (0.05 mM). Total RNA isolation was done with The RNEasy Midi Kit (Qiagen, Valencia, CA, United States). The Illumina Tru-seq RNA sample prep kit was used for targeted analysis of polyadenylated transcripts, Illumina HiSeq2500 was used for paired-end sequencing.

The FASTQ data files from the paired-end sequencing analysis were aligned with TopHat 2 (Kim et al., 2013) against the human GRCh38 reference genome assembly via the R package. The following RNAseq data quality control was carried out: 1) RNA quality; 2) raw read data (FASTQ); 3) alignment; 4) gene expression (Sheng et al., 2017). Quality control of the raw data and alignment was achieved with QC3 (Guo et al., 2014a), and the MultiRankSeq method was performed for expression analysis (Guo et al., 2014b). Raw data false discovery rate (FDR <0.05) was used to correct for multiple hypothesis testing.

### Statistical analysis

Data are provided as the mean and standard error of the mean (SEM) for continuous variables and as frequencies and percentages for categorical variables. In the analysis of IsoLG + APCs, the baseline percentages of immune cells containing IsoLGs and changes in these percentages in response to salt loading or salt depletion were used. Wilcoxon rank-sum test was utilized to compare the blood pressure changes from baseline to salt loading, and from salt loading to depletion. The associations between continuous variables of interest were assessed by Spearman’s rank correlation. Trend lines and confidence intervals (CI) were estimated using linear regression. A *p*-value less than 0.05 was determined to reject the null hypothesis. The Stata software version 16 was used in biostatistical analysis. GraphPad Prism version 9 was used to construct graphics, trend lines and confidence intervas of linear regression.

## Results

### Changes in renin-angiotensin system-associated genes in human monocytes in response to *ex vivo* high sodium exposure

**FIGURE 1:**
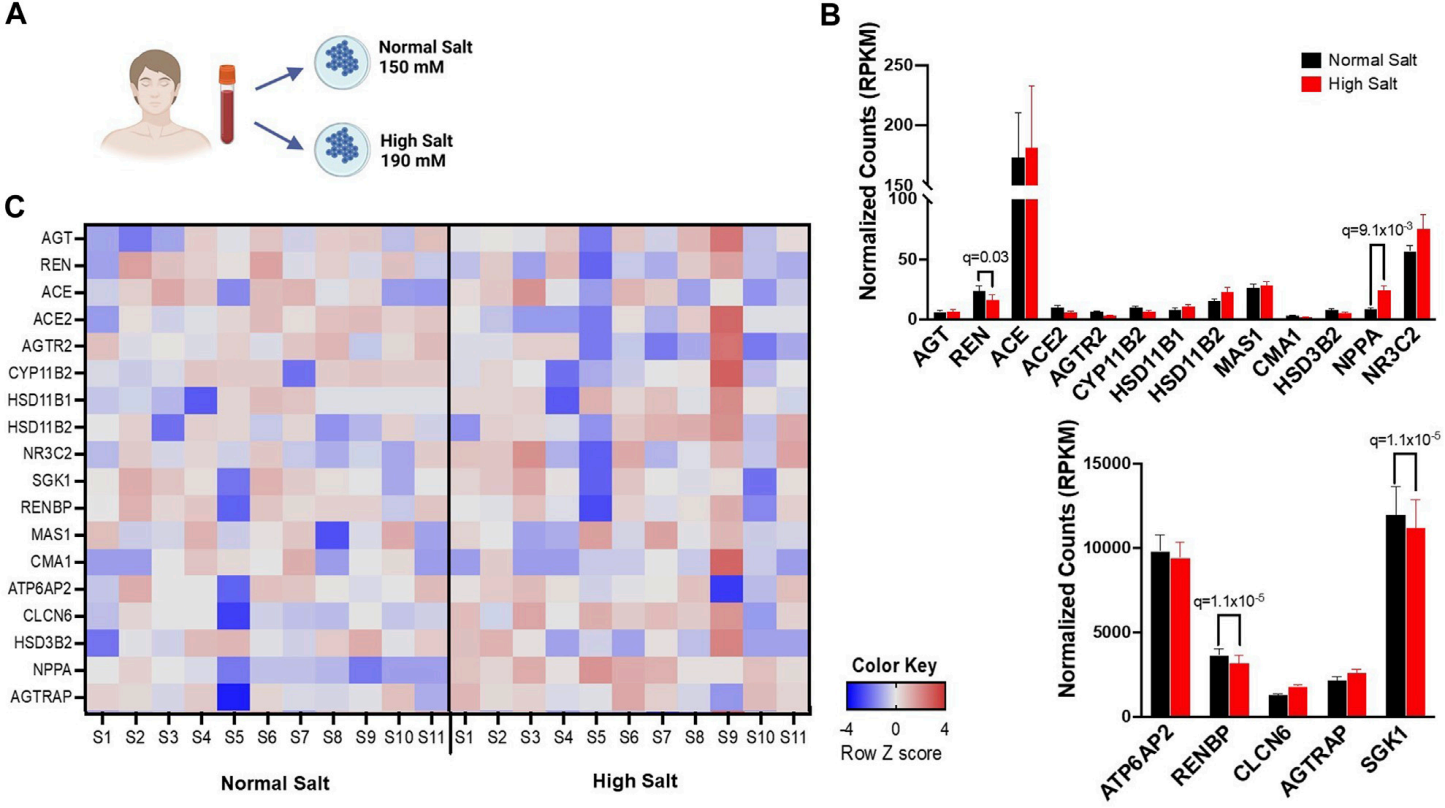
**(A)** Experimental design for bulk RNA sequencing. Human monocytes from 11 healthy subjects were isolated and cultured in either high salt (190 mmol/L NaCl) or normal salt (150 mmol/L NaCl) for 72 h **(B)**, Expression differences in RAS-related genes in response to normal salt and high salt treatment are presented in reads per kilobase per million mapped reads (RPKM). **(C)** A heatmap of RNA transcripts illustrating RAS-related gene expression differences in response to normal salt and high salt treatment is provided. Bright pink, bright blue, and white represent the highest, lowest, and median read values, respectively. Rows represent individual genes and columns represent individual samples. Panel A created with BioRender.com.

In recent studies, we found that human monocytes are activated by high concentrations of extracellular sodium (Ruggeri Barbaro et al., 2021). In order to explore the effects of high salt on the expression of RAAS-related genes, we employed bulk RNA sequencing on human monocytes extracted from eleven healthy subjects, whose demographics have been previously published (Pitzer et al., 2022) and depicted in Table 1. The monocytes were exposed to either normal (150 mMol/L) or high sodium (190 mMol/L, equivalent to high extracellular skin sodium concentrations observed *in vivo* (Titze et al., 2004)) for 72 h, as outlined in Figure 1A.

**TABLE 1:** Demographics of study participants presented in Figure 1.

Characteristics	Study population (*n* = 11)
Age (years)	34.7 ± 11.8
Female sex, n (%)	11 (100)
White race, n (%)	10 (91)
BMI (kg/m^2^)	28.8 ± 17.5
SBP (mmHg)	110.9 ± 16.7
DBP (mmHg)	69.0 ± 6.8
HTN, %	9

BMI, body mass index; DBP, diastolic blood pressure; SBP, systolic blood pressure. Data for continuous variables are presented as mean ± SEM.

We have previously found that high sodium *ex vivo* increased expression of genes associated with inflammatory activation including interleukin-1β (IL-1β), IL18 and inflammatory caspases (Pitzer et al., 2022). The expression of genes encoding angiotensinogen (*AGT*), renin (*REN*), angiotensin-converting enzyme (*ACE*), angiotensin-converting enzyme 2 (*ACE2*), angiotensin II receptor type 2 (*AGTR2*), aldosterone synthase (*CYP11B2*), hydroxysteroid 11-beta dehydrogenase 1 (*HSD11B1*), hydroxysteroid 11-beta dehydrogenase 2 (*HSD11B2*), nuclear receptor subfamily 3 group c member 2 (*NR3C2*), serum/glucocorticoid-regulated kinase 1 (*SGK1*), renin binding protein (*RENBP*), MAS1 G Protein-Coupled Receptor (*MAS1*), Chymase 1 (*CMA1*), ATPase H+ transporting accessory protein 2 (*ATP6AP2*), chloride transport protein 6 (*CLCN6*), hydroxy-delta-5-steroid dehydrogenase (*HSD3B2*), natriuretic peptide A (*NPPA*) and angiotensin II receptor associated protein (*AGTRAP*) were assessed. A representative heatmap including the expression of RAAS-associated genes is given in Figure 1C. *RENBP, ATP6AP2, CLCN6* and *AGTRAP* were highly expressed in monocytes in both normal and high salt condition. Exposure to high salt significantly downregulated the transcription of renin (*REN*), renin binding protein (*RENBP*), renin receptor (*ATP6AP2*), and upregulated the transcription of Natriuretic Peptide A (*NPPA*) (Figure 1B).

### Expression of renin-angiotensin system -associated genes in human myeloid immune cells in response to changes in dietary salt

**FIGURE 2:**
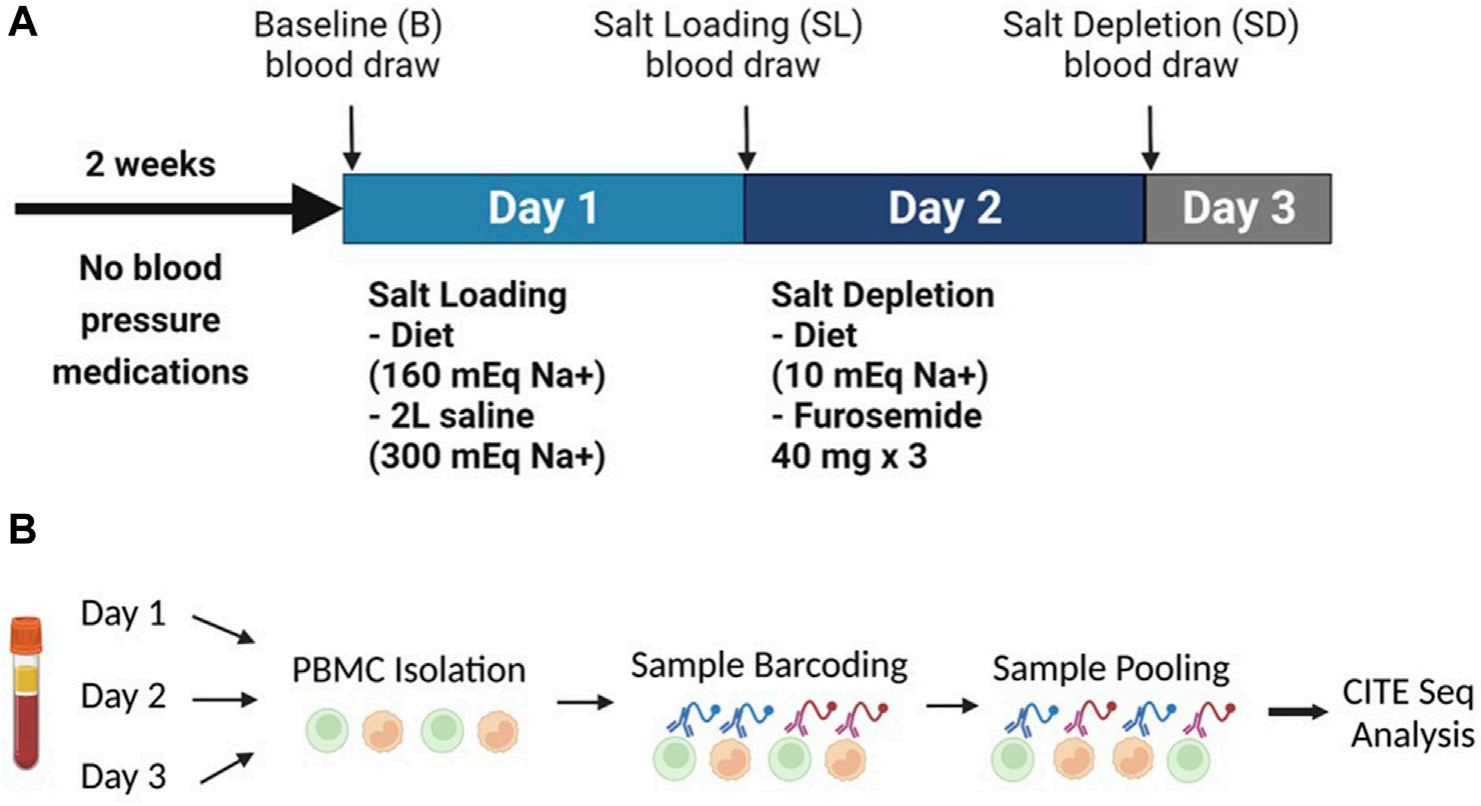
**(A)** The experimental design showing the inpatient protocol of salt loading (day 1) and salt depletion (day 2). **(B)** Experimental setup of the human peripheral blood mononuclear cells CITE-Sequence Analysis. Panel B created with BioRender.com.

To determine whether *in vivo* acute changes in dietary sodium dynamically regulate expression of RAAS-related genes in human immune cells and examine the association of such response with salt sensitive hypertension, we studied nine people with hypertension after salt loading and depletion with CITE-Sequence analysis. The study protocol is illustrated in Figures 2A, B and the demographic characteristics of the study patients, as previously published (Pitzer et al., 2022), are provided in Table 2.

**TABLE 2:** Demographics of study participants presented in Figure 2.

Characteristics	Study population (n = 9)
Age (years)	50.9 ± 2.1
Male sex, n (%)	6 (66.7)
White race, n (%)	9 (100)
BMI (kg/m^2^)	34.5 ± 3.9
Screening SBP (mmHg)	139.1 ± 4
Screening DBP (mmHg)	87.7 ± 3.9
Plasma renin concentration (ng/L)	15.0 ± 6.1
Plasma aldosterone (ng/dL)	14.7 ± 4.0

BMI, body mass index; DBP, diastolic blood pressure; SBP, systolic blood pressure. Data for continuous variables are presented as mean ± SEM.

**FIGURE 3:**
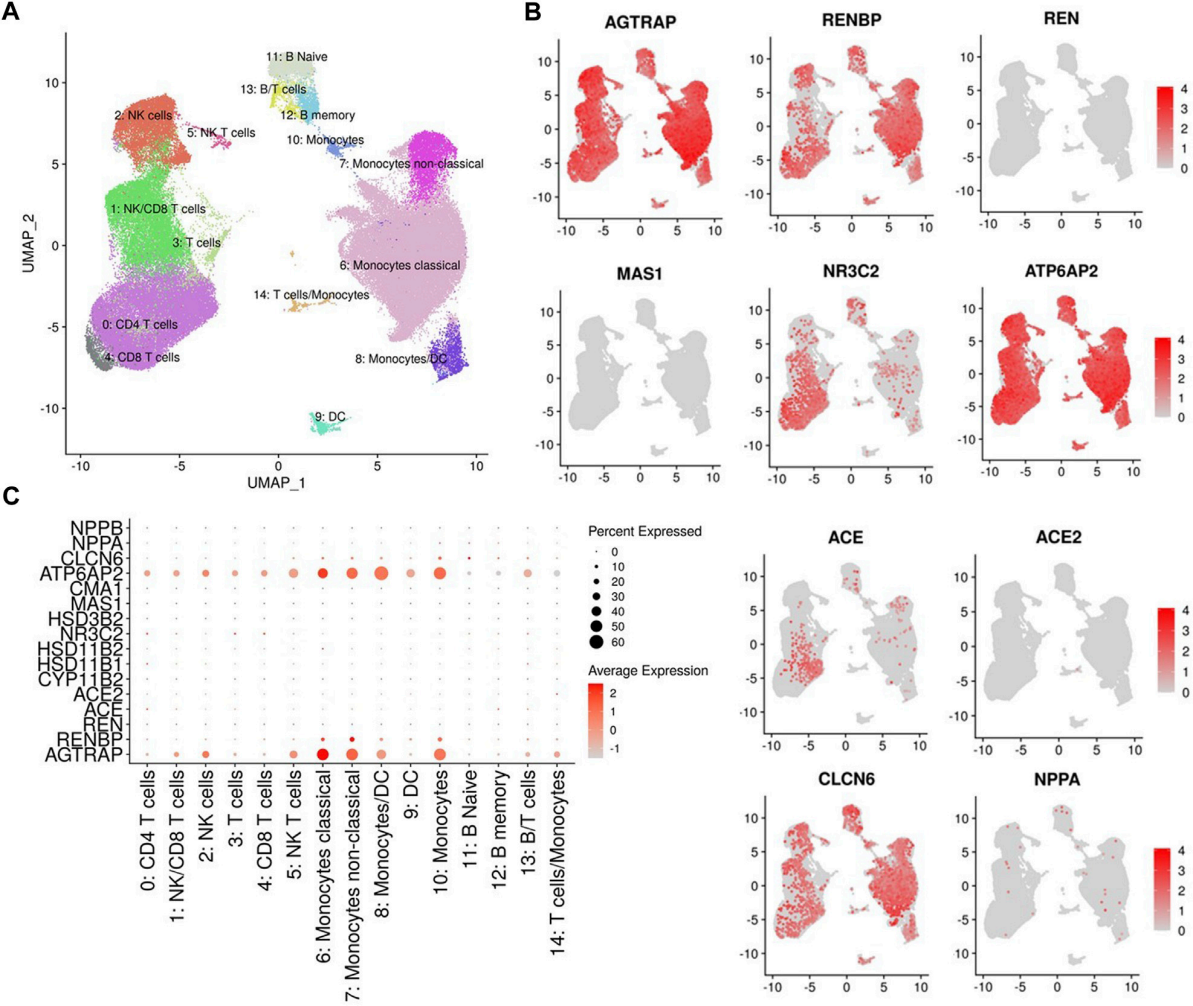
**(A)** UMAP representation of immune cell type clusters identified by antibody-derived tags. **(B)** Gene expression of Angiotensin II Receptor Associated Protein (AGTRAP), Renin Binding Protein (RENBP), Renin (REN), MAS1 proto-oncogene (MAS1) which encodes angiotensin-(1-7) receptor, Nuclear receptor subfamily 3 group c member 2 (NR3C2) which encodes the mineralocorticoid receptor, ATPase H+ transporting accessory protein 2 (ATP6AP2) which encodes the (pro)renin receptor, angiotensin I converting enzyme (ACE), angiotensin I converting enzyme 2 (ACE2), Chloride transport protein 6 (CLCN6), and Natriuretic Peptide A (NPPA) in different cell clusters are presented in UMAPs. **(C)** Dot plot of RAAS-related gene expressions in different cell clusters.

To investigate dietary salt-induced *in vivo* changes in transcription of RAAS-related genes in immune cells, we used cell hashing and Cellular Indexing of Transcriptomes and Epitopes by Sequencing analysis to profile the transcriptomes of PBMCs at baseline, salt loading and salt depletion. The UMAP clustering of different immune cell types is provided in Figure 3A. We found that angiotensin II receptor-associated protein *AGTRAP* and renin receptor *ATP6AP2* are widely expressed in all cell clusters (Figure 3B) with greater expression in monocytes and dendritic cells (Figure 3C). Renin binding protein, encoded by *RENBP*, was found to be expressed predominantly in monocytes and dendritic cells (Figure 3C). *ACE* expression was found in T cells but not in monocytes and dendritic cells, as previously reported (Figure 3B) (Costerousse et al., 1993). Corroborating previous findings (Song et al., 2023), we found no expression of *ACE2* in circulating immune cells (Figure 3B). Also, there was no expression of *MAS1* and *REN* genes in circulating immune cells (Figure 3B). Mineralocorticoid receptor, encoded by *NR3C2*, was expressed predominantly in T cells, with low levels of expression in monocytes and dendritic cells (Figure 3B).

**FIGURE 4:**
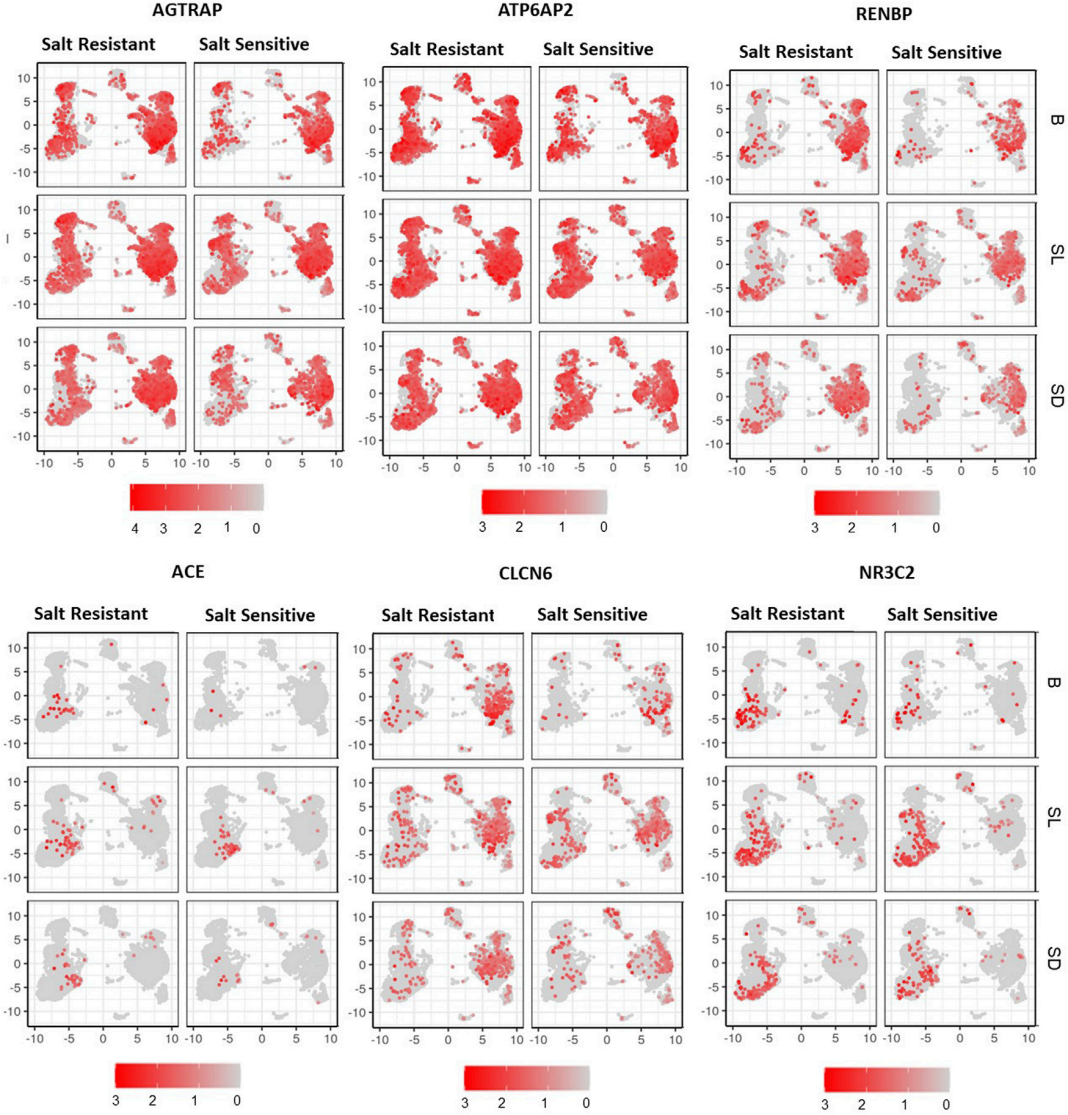
UMAP representation of gene expression of AGTRAP, ATP6AP2, RENBP, ACE, CLCN6, and NR3C2 are shown at baseline (B), salt loading (SL) and salt depletion (SD) in select salt resistant and salt sensitive subjects.

Figure 4 demonstrates the expressions of *AGTRAP*, *ATP6A2*, *RENBP, ACE, NR3C2*, and *CLCN6* in cell clusters from salt sensitive versus salt resistant subjects at baseline, salt loading and salt depletion. There was a trend of decreased expression of *AGTRAP*, *ATP6A2*, and *RENBP* from salt loading to salt depletion in classical monocytes and dendritic cells of salt sensitive subjects. No statistically significant difference was found in the expression of any RAAS-related gene between salt loading and depletion. Among the T cell clusters, AGTRAP and NR3C2 expressions demonstrated an increasing trend with salt loading and decreasing trend with salt depletion, which was more pronounced in salt sensitive patients. Similarly, the expression of RENBP in T and CK cell clusters showed an increasing trend from baseline to salt loading, and a decreasing trend from salt loading to depletion within salt sensitive patients.

**FIGURE 5:**
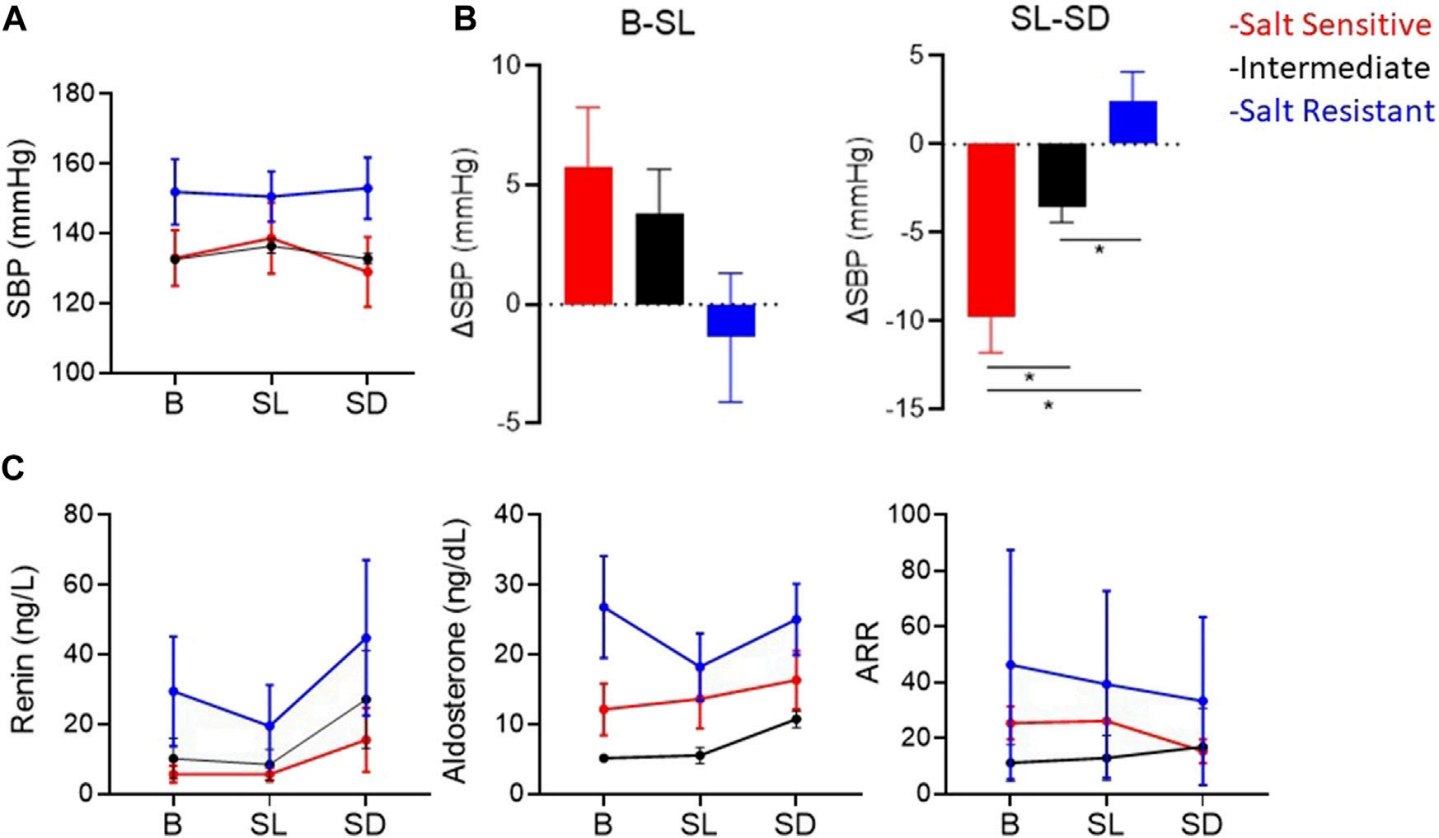
**(A)** Mean ± SEM of systolic blood pressures (SBP). **(B)** Mean ± SEM of the change in SBP from baseline (B) to salt loading (SL), and from salt loading to salt depletion (SD). *p < 0.05 assessed by Wilcoxon rank-sum test. **(C)** Mean ± SEM of plasma renin concentration, plasma aldosterone, and aldosterone/renin ratio (ARR) atB, SLand SD. The red, blue and black lines represent the patients with the highest, the lowest and intermediate salt sensitivity index, respectively.

**FIGURE 6:**
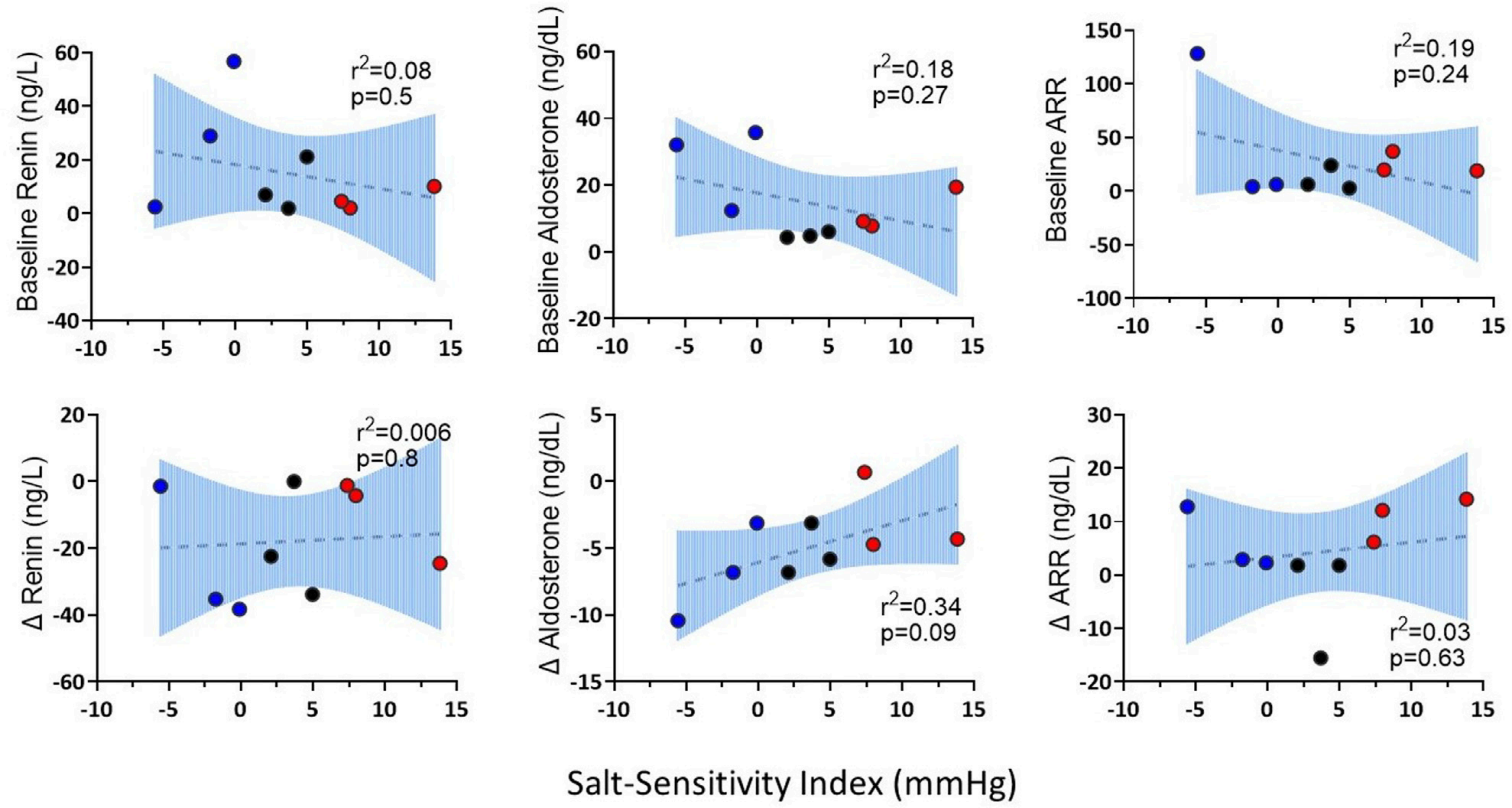
The correlations of baseline renin, aldosterone, ARR and changes (∆) in renin, aldosterone and ARR from salt loading to salt depletion (SL-SD) with salt sensitivity index. Three patients with the highest salt sensitivity index were represented as salt sensitive (red); three patients with intermediate salt sensitivity index were represented as intermediate (black); three patients with the lowest salt sensitivity index were represented as salt resistant (blue).

Figure 5 shows the SBP, plasma renin and aldosterone concentrations and aldosterone/renin ratio (ARR) of patients with highest, intermediate and lowest salt sensitivity index at baseline and after salt loading and depletion. The changes in SBP from salt loading to depletion were significantly different between the groups (Figures 5A, B). We observed that baseline renin and aldosterone levels of salt sensitive subjects tended to be lower with a blunted response to the salt loading and depletion challenge (Figure 5C), as previously reported (He et al., 2001; Yatabe et al., 2010). The relationships between levels of renin and aldosterone and salt-sensitivity index were not statistical significant (Figure 6).

## Discussion

In this study, we investigated the effects of high levels of sodium *in vitro* and dietary salt intake on the transcription of RAAS-regulatory genes in myeloid immune cells. Our findings suggest that salt-induced myeloid immune cell activation is modulated independent of RAAS-related pathways. We further report that *in vivo* changes in immune cell expression of RAAS-associated genes in response to salt loading and depletion are similar in salt sensitive and salt resistant people.

In previous animal studies, we established a pathway by which sodium enters APCs via amiloride-sensitive ENaC, which activates NAPDH oxidase, and results in lipid peroxidation and IsoLG-adduct formation. IsoLG-adducts are highly immunogenic and lead to immune cell activation, salt-sensitive hypertension, and end-organ damage (Kirabo et al., 2014; Barbaro et al., 2017). While our previous findings established salt-sensing kinase serum/glucocorticoid kinase 1 (SGK1) as a promoter of ENaC activity in APCs (Van Beusecum et al., 2019), the mechanisms by which the ENaC-dependent IsoLG response is regulated differently in salt-sensitive and salt-resistant people largely remain unknown. Our current findings suggest that RAAS does not play a major role in the regulation of salt-induced myeloid immune cell activation in salt sensitive hypertension. Further studies would be needed to reveal the mechanistic pathways by which salt-sensitivity associated ENaC activity and IsoLG-adduct formation are modulated in APCs.

We found that *ex vivo* exposure of human monocytes from healthy subjects to high salt significiantly downregulated the expression of *REN* and *RENBP,* encoding rening and renin binding protein. Although no significant changes were found in the expression of either gene in circulating myeloid immune cells with *in vivo* changes in dietary salt, there was a decreasing trend in the expression of *RENBP* with salt depletion in antigen presenting cells of salt sensitive hypertensive subjects. While marginal expression of *REN* was detected *ex vivo*, there was no detectable expression of *REN* in immune cells *in vivo*. Further studies are required to investigate whether the expression of *RENBP* in myeloid immune cells may be regulated by salt in salt-sensitivite people.

CITE-Sequence analysis revealed very low transcription of the mineralocorticoid receptor gene in monocytes and dendritic cells at both salt loaded and depleted states, suggesting the absence of aldosterone regulation of immune cell ENaC in salt sensitivity.

RAAS is a critical regulator of blood pressure and renal sodium balance. While salt sensitivity is characterized by diminished RAAS response to changes in dietary salt (Parfrey et al., 1981; Laffer et al., 2003; Elijovich et al., 2016) as also observed in the current study, the exact role of RAAS in the pathogenesis of salt sensitive hypertension is incompletely understood. Our findings suggest that RAAS-related gene expression in myeloid immune cells is not regulated by acute changes in dietary salt intake.

A major limitation of the current study is the small sample size. While there was a trend of a blunted renin-angiotensin system in the salt sensitive patients as published previously (He et al., 2001; Yatabe et al., 2010), the small sample size may have precluded the detection of a statistically significant relationship between plasma renin and aldosterone response and salt sensitivity index. Moreover, since women are affected by salt sensitivity more (Sahinoz et al., 2021), further studies with large sample sizes are required to study potential gender differences in the regulation of RAAS in myeloid immune cells in salt sensitivity. It should also be noted that the study population ofthe salt loading and depletion protocol lacked racial diversity. Given that salt sensitivity is more commonly found in blacks (Sahinoz et al., 2021), potential racial differences should be investigated in future studies. Nevertheless, we provide the first exploratory findings that investigate the effects of acute dietary salt loading and depletion on RAAS-related gene expression in myeloid immune cells. The study sample of the CITE-Seq analysis excludes people without hypertension which limits the application of these findings to salt sensitive people without hypertension. Since the inpatient protocol derived by Weinberger et al. (1986) depicts acute effects of sodium only, studies involving longer periods of high salt and low salt are needed to assess chronic effects of sodium.

In conclusion, our findings suggest that the expression of RAAS-related genes do not associate with salt-induced myeloid immune cell activation in salt sensitive hypertension. Further studies are needed to reveal the mechanisms by which salt-induced APC activation is regulated, which may offer therapeutic options to inhibit subsequent inflammatory response and end-organ damage.

## Data Availability

The data has been previously published (doi: 10.1161/CIRCRESAHA.122.320818) and uploaded to Figshare, and is available at https://figshare.com/s/d810937dc537eeb361a5.
